# Fast field-cycling magnetic resonance detection of intracellular ultra-small iron oxide particles *in vitro*: Proof-of-concept

**DOI:** 10.1016/j.jmr.2020.106722

**Published:** 2020-04

**Authors:** Hassan Abbas, Lionel M. Broche, Aiarpi Ezdoglian, Dmitriy Li, Raif Yuecel, P. James Ross, Lesley Cheyne, Heather M. Wilson, David J. Lurie, Dana K. Dawson

**Affiliations:** a Aberdeen Cardiovascular and Diabetes Centre, University of Aberdeen, Aberdeen, United Kingdom; b Bio-Medical Physics, School of Medicine, University of Aberdeen, Aberdeen, United Kingdom; c Iain Fraser Cytometry Centre, Institute of Medical Sciences, University of Aberdeen, Foresterhill, Aberdeen AB25 2ZD, United Kingdom; d Department of Medical Chemistry and Toxicology, NI Pirogov Russian National Research Medical University, Moscow 117997, Russian Federation^1^; e Cytomics Centre, College of Life and Environmental Sciences, University of Exeter, EX4 4QD, United Kingdom^1^

**Keywords:** Fast field-cycling magnetic resonance, Inflammation, Ultrasmall superparamagnetic iron oxide particles (USPIO), FC-MRI, Fast Field-Cycling Magnetic Resonance Imaging, FFC-NMR, Fast Field-Cycling Nuclear Magnetic Resonance, MRI, Magnetic Resonance Imaging, SSC, Side Scatter, USPIO, Ultra-Small Superparamagnetic Iron Oxide, SPIO, Superparamagnetic Iron Oxide

## Abstract

**Purpose:**

Inflammation is central in disease pathophysiology and accurate methods for its detection and quantification are increasingly required to guide diagnosis and therapy. Here we explored the ability of Fast Field-Cycling Magnetic Resonance (FFC-MR) in quantifying the signal of ultra-small superparamagnetic iron oxide particles (USPIO) phagocytosed by J774 macrophage-like cells as a proof-of-principle.

**Methods:**

Relaxation rates were measured in suspensions of J774 macrophage-like cells loaded with USPIO (0–200 μg/ml Fe as ferumoxytol), using a 0.25 T FFC benchtop relaxometer and a human whole-body, in-house built 0.2 T FFC-MR prototype system with a custom test tube coil. Identical non-imaging, saturation recovery pulse sequence with 90° flip angle and 20 different evolution fields selected logarithmically between 80 μT and 0.2 T (3.4 kHz and 8.51 MHz proton Larmor frequency [PLF] respectively). Results were compared with imaging flow cytometry quantification of side scatter intensity and USPIO-occupied cell area. A reference colorimetric iron assay was used.

**Results:**

The *T*_1_ dispersion curves derived from FFC-MR were excellent in detecting USPIO at all concentrations examined (0–200 μg/ml Fe as ferumoxytol) vs. control cells, *p* ≤ 0.001. FFC-NMR was capable of reliably detecting cellular iron content as low as 1.12 ng/µg cell protein, validated using a colorimetric assay. FFC-MR was comparable to imaging flow cytometry quantification of side scatter intensity but superior to USPIO-occupied cell area, the latter being only sensitive at exposures ≥ 10 µg/ml USPIO.

**Conclusions:**

We demonstrated for the first time that FFC-MR is capable of quantitative assessment of intra-cellular iron which will have important implications for the use of USPIO in a variety of biological applications, including the study of inflammation.

## Highlights

• FFC-MR can detect and quantify dependence of macrophage USPIO *R*_1_ dispersion *in vitro*.
• Inflammation assessment *in vivo* and the utility of USPIO may be enhanced by FFC-MR.
• Accessing tissue *T*_1_ dispersion properties may complement FFC-MR imaging techniques.

## Graphical abstract

**Figure:**
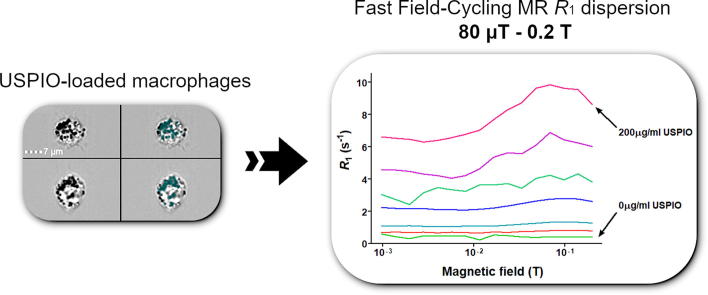

## 1 Introduction

Tissue inflammation is a recognised disease process in many cardiovascular conditions, whether it involves the myocardium or the arterial vascular wall [1]. Inflammatory cell tracking with imaging *in vivo* is likely to become part of an individualised diagnostic pathway, assessing the severity of disease and monitoring the response to anti-inflammatory therapies. Inflammatory macrophages are particularly well suited for MR imaging due to their strong phagocytic ability to uptake contrast agents such as ultra-small superparamagnetic iron oxide (USPIO) particles [2]. Phagocytosed USPIOs are retained in inflammatory cells after the intra-vascular pool is cleared. Due to their superparamagnetic properties, USPIOs internalised by macrophages can be tracked with Magnetic Resonance Imaging (MRI) techniques *in vivo* [3]. The dipolar relaxivity describes the effect a contrast agent on proton relaxation rate in a homogenous medium, and is defined by

*R*_1,2_ = *R*^0^_1,2_ + *r*_1,2_. *C* (where *R*_1,2_ is the *R*_1_ or *R*_2_ proton relaxation rate in the presence of the contrast agent. *R*^0^_1,2_ are the relaxation rates in the absence of the contrast agent, *r*_1,2_ are the relaxivities in s^−1^ mM^−1^ and *C* is the contrast agent concentration) [4].

USPIOs have a 10-fold (or higher) stronger effect on T2 relaxivity than gadolinium in clinical scanners [4] and have potential for detection at much lower concentration. They create large magnetic field distortions which affect nearby water molecules and shorten *T*_2_* therefore creating regions of signal loss [5]. Detection has been achieved at routine clinical magnetic fields (1.5 and 3 T) using *T*_2_* sequences [6]. *T*_1_-relaxivity can also be higher than in gadolinium, which in turn can vary as a function of field strength, core size and degree of particle aggregation (15–25 s^−1^ mM^−1^ in water at clinical field range) [7], [8]. By comparison the *r*_1_ of gadolinium chelates is fairly constant at clinical field strengths (approx. 4 s^−1^ mM^−1^). Our group has previously imaged myocardial inflammation in humans using USPIO during acute stress-induced cardiomyopathy [9]. Uptake of USPIO in infarcted myocardium has been previously demonstrated in a clinical experiment with focus on *T*_2_* effects at 3 T [10]. Patients with distinct mural uptake of USPIO within abdominal aortic aneurysms (AAA) had a 3-fold higher growth rate, suggesting that cellular inflammation may predict AAA expansion and thus risk of rupture [11]. Similarly USPIO-enhanced MRI of carotid plaque can be used to detect ruptured or rupture-prone atherosclerotic lesions [12], [13].

Experiments on infarcted human myocardium have shown that *R*_2_* (1/*T*_2_*) increases from a baseline of 41.0 ± 12.0 s^−1^ to 155 ± 45.0 s^−1^ at 3 T, 24 h following administration of USPIO, which demonstrates an extremely short *T*_2_* with a narrow dynamic range in this application (approx. 6–24 ms) [10].

There has been a tendency over the years to increase the magnetic fields strengths to enhance image resolution and improve signal-to-noise ratio (SNR). However, the *T*_1_ values of different tissues tend to converge at higher field strengths (>1.5 T), causing the endogenous *T*_1_ contrast to diminish [14]. The relaxivity of most exogenous contrast agents used to overcome this problem is also dependent on magnetic field strength, tending to be lower at higher field strengths [15], [16], [17], [18], [19]. USPIO may therefore add better value in low-field imaging systems. Therefore, an exploration into the field-dependency of USPIO contrast in biological media becomes a prerequisite to targeting low-field MRI devices for these applications. The use of USPIO as a contrast agent in low field MRI has only been recently reported [20] and in such regimes USPIOs are efficient *T*_1_ contrast agents thanks to their superparamagnetic properties [19]. Yin et al have more recently demonstrated strong *T*_1_ enhancement with 18 nm diameter USPIO at ultra-low field (0.13mT), with longitudinal relaxivity in solution (deionised water) of *r*_1_ = 615 s^−1^ mM^−1^, two orders of magnitude higher than typical Gd-based preparations at clinical fields (1.5–3 T) [21]. The enhanced *R*_1_ at low field was attributed to the coupling of proton spins with SPIO nanoparticle magnetic fluctuations (Brownian and Néel) with a low frequency peak in the imaginary part of AC susceptibility (χ″). Therefore, a low magnetic field device may have distinct advantages in assessing the presence and intensity of the inflammatory response, by accurately detecting and quantifying the uptake of such inflammation-tracking USPIO contrasts. The high *T*_1_ signal at low field may also reduce imaging time and the non-toxic, biocompatibility of USPIO may be more welcome than nephrotoxic Gd-based agents.

Here we propose to exploit USPIOs as a *T*_1_ contrast agent for tissue inflammation using Fast Field-Cycling Nuclear Magnetic Resonance (FFC-NMR) at magnetic fields below 0.25 T in an effort to better understand how this contrast agent may be used in low-field systems. Beyond the advantages of a fixed low magnetic field, a *field-cycling* device may also allow the discrimination of USPIO contribution from other sources of relaxation due to its ability to delineate and quantify the ferromagnetic peak on the 1/*T*_1_ dispersion profile of USPIO [22], [23].

Our group is at the forefront of developing Fast Field-Cycling Magnetic Resonance Imaging (FFC-MRI) for medical applications [24], [25]. FFC-MRI is an imaging modality using the principles of FFC-NMR, which measures the longitudinal nuclear magnetic resonance relaxation time (*T*_1_) as a function of the magnetic field strength (*B*_0_) and allows access to *T*_1_ over a range of magnetic fields. The systems used in our labs allow accessing low magnetic fields from 20µT to 250 mT. These measurements are then processed to provide the field dispersion of *R*_1_ (where *R*_1_ = 1/*T*_1_), also called the *R*_1_ dispersion profile. It is well-known that *R*_1_ varies greatly between biological tissues [26], [27] and can therefore be exploited for diagnostic applications. FFC-NMR pulse sequences typically comprise three periods, polarisation, evolution and detection, during which different magnetic fields are applied (*B*_0_^P^, *B*_0_^E^ and *B*_0_^D^ respectively). Polarisation increases the signal amplitude for improved contrast, evolution generates the field-dependant evolution that is needed to extract *R*_1_ dispersion information and detection is used to measure the NMR signal to be processed. Crucially, signal detection measurements are always carried out at the same magnetic field, *B*_0_^D^, so that the resonant frequency γ*B*_0_^D^ remains the same throughout the experiment (where γ is the gyromagnetic ratio of the nuclear spin system studied).

In this work we hypothesized that FFC-NMR is capable of detecting and quantifying the superparamagnetic signal of USPIO phagocytosed by cells with a sensitivity comparable to validated techniques of iron quantification in biological samples, such as standard colorimetric assays. We also compare FFC-NMR with the imaging flow cytometric features of USPIO-laden cells.

## 2 Methods

### 2.1 Cell preparation

Murine BALB/c J774A.1 macrophage-like cells (Sigma-Aldrich, ECACC code 91051511, no mycoplasma detectable microscopically) were cultured in DMEM (Gibco, Cat # 11960044) supplemented with 1 mM L-glutamine (Gibco, Cat # 25030081), 1% penicillin/streptomycin (Gibco, Cat # 15140122) and 10% foetal bovine serum (Gibco, Cat # 12676029) at 37 °C with 5% CO_2_. For iron loading, cells plated at 1x10^6^ cells/well in triplicates, 6-well plates (Thermo Scientific, Cat # 14067) were incubated with 0 (control), 5, 10, 40, 80, 100 and 200 µg/ml Fe as USPIO (ferumoxytol, Rienso, core diameter 10.8 ± 1.8 nm, hydrodynamic diameter 30.8 ± 0.1 nm) for 16 hrs. Concentrations expressed are of Fe contained within USPIO and do not represent concentrations of particles. The J744 macrophages were washed three times with PBS (BioWhittaker, Cat # 17516F) to remove non-phagocytosed USPIO’s, collected by cell scraping and resuspended in 500 µl PBS/2mM EDTA (BioWhittaker, Cat # 17-711E). Experiments utilising fresh cells were conducted immediately following PBS/EDTA suspension, the samples transported on ice and light protected. For fixation experiments, cells were washed and scraped as above then fixed in 4% paraformaldehyde (Sigma-Aldrich, Cat # HT501128-4L) for 15mins on ice. The cells were then spun, washed twice with PBS then re-suspended in 500 µl PBS/2mM EDTA and analysed within 24hrs (light-protected and stored at 4 °C). Samples for all experiments were prepared in triplicate. J774 phagocytosis was confirmed on light microscopy (EVOS XL Core, USA) using microbeads (Polysciences Inc., Cat # 18133-2) and USPIO internalisation using a Perl iron stain kit (TCS Biosciences, Cat # HS652) yielding Prussian blue.

### 2.2 Fast field-cycling nuclear magnetic resonance experiments

Relaxation rate measurements were carried out using both a 0.25 T FFC-NMR benchtop relaxometer (SMARtracer, Stelar, Italy) and a human whole-body, in-house built prototype 0.2 T FFC-MRI system [24] which was used as a relaxometer (University of Aberdeen EU Horizon 2020 programme). The RF coil used was a home-built transmit/receive solenoid with an inner diameter of approximately 10 mm, tuned to 8.5 MHz. Cell suspensions were prepared as above to a total volume of 500 µl PBS/2mM EDTA and analysed in glass test tubes (Samco, Cat # G050/18) at 21 °C. Both devices produced the same quantification of *T*_1_ and therefore the results were comparable. On both devices*, R*_1_ dispersion measurements were conducted using an identical non-imaging, saturation recovery pulse sequence with 90° flip angle and 20 different evolution fields selected logarithmically between 80 μT and 0.2 T (3.4 kHz proton and 8.51 MHz Larmor frequency [PLF] respectively). The evolution times were modified for each sample and field, using 12 different values selected logarithmically from 0.1 to 4 times the *T*_1_ obtained from the previous measurement. The acquisition bandwidth was set to 20 kHz for the FFC-MRI system and 80 kHz for the relaxometer to account for lower field homogeneity, and in both cases the signal was recorded until it reached the noise floor (Free Induction Decay (FID) length, approximately 50 ms for the FFC-MRI system and 5 ms for the relaxometer). In total, the measurement time was less than 35 min per sample. *R*_1_ of the test sample was measured at each evolution field, for each of the triplicates. The average *R*_1_ of the three samples was then calculated for each evolution field, and reported for each USPIO exposure. The effect of USPIO exposure was measured simply by averaging the dispersion curve at each concentration, in comparison with that of the control cell suspensions, which did not show any *R*_1_ dispersion and could therefore be treated as a constant baseline of approximately 0.3 s^−1^ in the *R*_1_ dispersion profiles. The dispersion curves obtained were analysed from 1.4 mT to 200 mT, a range that provided robust measurements on both the benchtop and whole-body FFC devices. Spurious noise occasionally generated gross outliers (These appeared at evolution fields below 200μT (5 kHz PLF) due to the presence of non-homogeneous environmental external magnetic fields), which were removed manually before the triplicate measurements were averaged and the contribution of non-USPIO relaxation was subtracted from the data by subtracting the average relaxation rate of the USPIO-free samples.

Since models are not readily available for USPIO relaxivity in biological tissues, we compared the data to a reference *T*_1_ dispersion curve. This reference was obtained for each dataset (fresh or fixed) by isolating the dispersion curve measured with the signal with best SNR, and subtracting from it the average of the dispersion curve obtained for solutions without USPIO, in order to remove the contribution of non-USPIO relaxation pathways. This approach relies on the preservation of the shape of the dispersion curves for increasing concentrations of USPIO, which is known to be an approximation but is fit for the purpose of this proof-of-concept work. Once the reference was obtained, it was compared to the dispersion curves of the samples by curve fitting, taking a multiplication factor as the optimisation factor. The quality of fit was measured using the Akaike Information Criterion (AIC) [28] which provided average values of AIC of −54 ranging from −92 to −20. This single-parameter analysis provided us with an estimate of the amplitude of the USPIO contribution at different exposures, which appeared exponential to a good approximation (AIC = −21 and −29 for fixed and fresh cell data respectively).

### 2.3 Imaging flow cytometry

Imaging flow cytometry was performed using the ImageStreamX Mark II (Luminex Corporation, USA), which combines the quantitative power of flow cytometry and imaging feature of microscopy, recording fluorescence intensities as well as bright-field (BF) images of individual cells. Fixed and non-fixed J774 cells were prepared as above and resuspended in 80 µl PBS/2mM EDTA (pH 7.5) and measurements obtained using the ImageStreamX Mark II INSPIRE acquisition software with magnification set to ×60. ImageStream data were analysed using the proprietary analysis software IDEAS supplied by the manufacturer. The software allows the exploration of series of image features derived from each single cell based on automatically generated masks for each imaging channel.

**Fig. 1:**
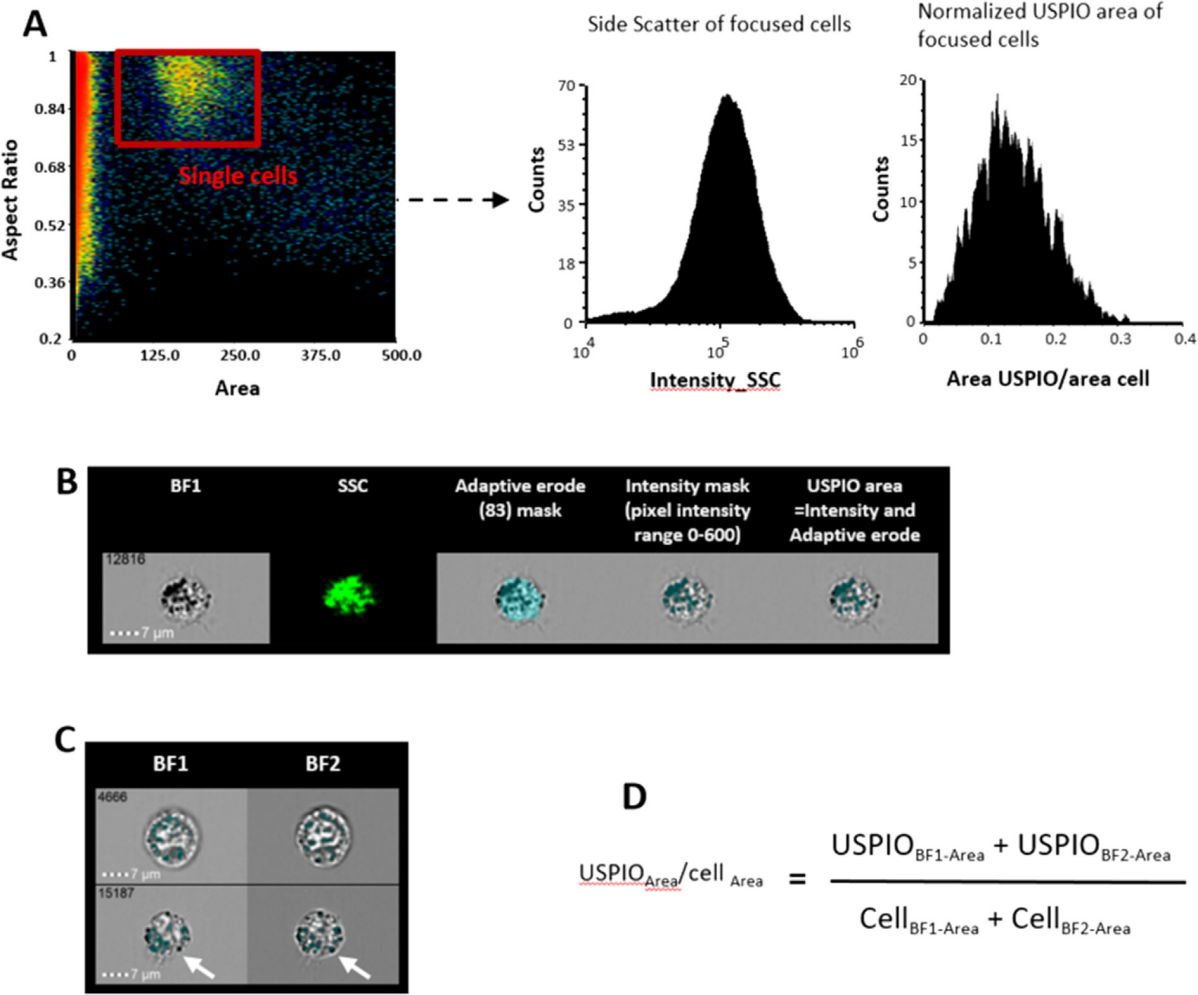
Outline of the workflow used to delineate area occupied by USPIO and cell granularity, based on calculated cellular features on the Amnis ImageStreamX Mark II. A: Single cells were selected by plotting area against aspect ratio assuming that most non-adherent macrophages were round in shape. Focused cells were selected by plotting gradient against contrast and selecting cells with highest values of both features. SSC was measured to assess granularity. B: Both custom-generated (green) and the ‘adaptive erode’ mask (no. 83) were used to identify USPIO-free intracellular area. USPIOs were identified using ‘intensity’ mask with the pixel intensity range (0–600). Intracellular USPIOs were identified by selecting overlapping cell- and USPIO-occupied areas. C: Images from two BF channels were included in the final calculation, given the slight difference between these images (white arrows). D: Calculation used to identify the relative areas of the cells occupied by USPIO. (For interpretation of the references to colour in this figure legend, the reader is referred to the web version of this article.)

Single macrophages were selected by plotting the area feature of brightfield channel 1 (BF1) versus the aspect ratio parameter of the same BF channel, which is the ratio of minor axis to the major axis of the applied mask and describes the shape of the mask applied to the cells (Fig. 1A). Focused cells were then selected by plotting the ‘Gradient RMS’ (RMS = root mean square) feature of BF1 against the contrast parameter of BF1. Cells with high gradient (>60) and high contrast value were more in focus and chosen for further analysis. Features of focused cells were analysed using custom-generated post-processing masks. Approximately 2000 focused cells were used in the analysis at each concentration.

Side Scatter (SSC) intensity was measured to assess cell granularity in non-fixed cells only (Fig. 1A). Additional quantification of USPIO-containing dark locules was performed using customised masks on two BF channels (Fig. 1C). The ‘Adaptive Erode’ mask on IDEAS then an ‘Intensity’ mask were applied to achieve this.

To exclude size variation between different cells, USPIO occupied area was normalized to the entire area of the cells. Therefore the area of the cells occupied by USPIO was calculated using the formula: (USPIO areaBF1 + USPIO areaBF2)/(CELL areaBF1 + CELL areaBF2) (Fig. 1D). The data was expressed as mean ± SEM of the relative area of the cell occupied by USPIO.

### 2.4 Colorimetric iron assay

Following plating, iron loading and washing as above, the PBS was removed and the plates were frozen at −20 °C pre-analysis. Triton X-100 (Sigma-Aldrich, Cat # T8787-50ML, 200 µl at 0.01%) solution was added directly to each well and the cells were harvested with a cell scraper. After micropipetting to encourage lysis, the cell lysates were transferred to microtubes. Cell lysate (10 µl) was removed from each sample for protein quantification using an improved Lowry [29] -type assay (BIO-RAD DC protein assay, Cat # 500-011).

A sensitive and validated colorimetric assay [30] utilising 2,2′-bipyridine (Fisher Scientific, Cat # 11492438) was calibrated and used to quantify total iron content in remaining cell lysate. A standard curve was constructed by adding 10 µl of 10 × 10^−3^, 6 × 10^−3^, 4 × 10^−3^, 1.6 × 10^−3^, 0.64 × 10^−3^, and 0.26 × 10^−3^ M freshly prepared solutions of ammonium iron (II) sulphate hexahydrate (Sigma-Aldrich, Cat # 203505) to ‘control’ cell lysate (no USPIO exposure). Absorbance at 520 nm was measured in a 96-well plate on a Synergy HT (BioTek Instruments Inc., USA) plate reader. Iron concentration was calculated by averaging triplicate samples, expressed as ng/µg protein.

### 2.5 Statistical analysis

All data is shown as mean ± SEM. Independent sample *t*-test was used to compare means, regression analysis to compare methods and Pearson *r for* correlations of data-sets. Statistical significance was set at *p* < 0.05.

## 3 Results

**Fig. 2:**
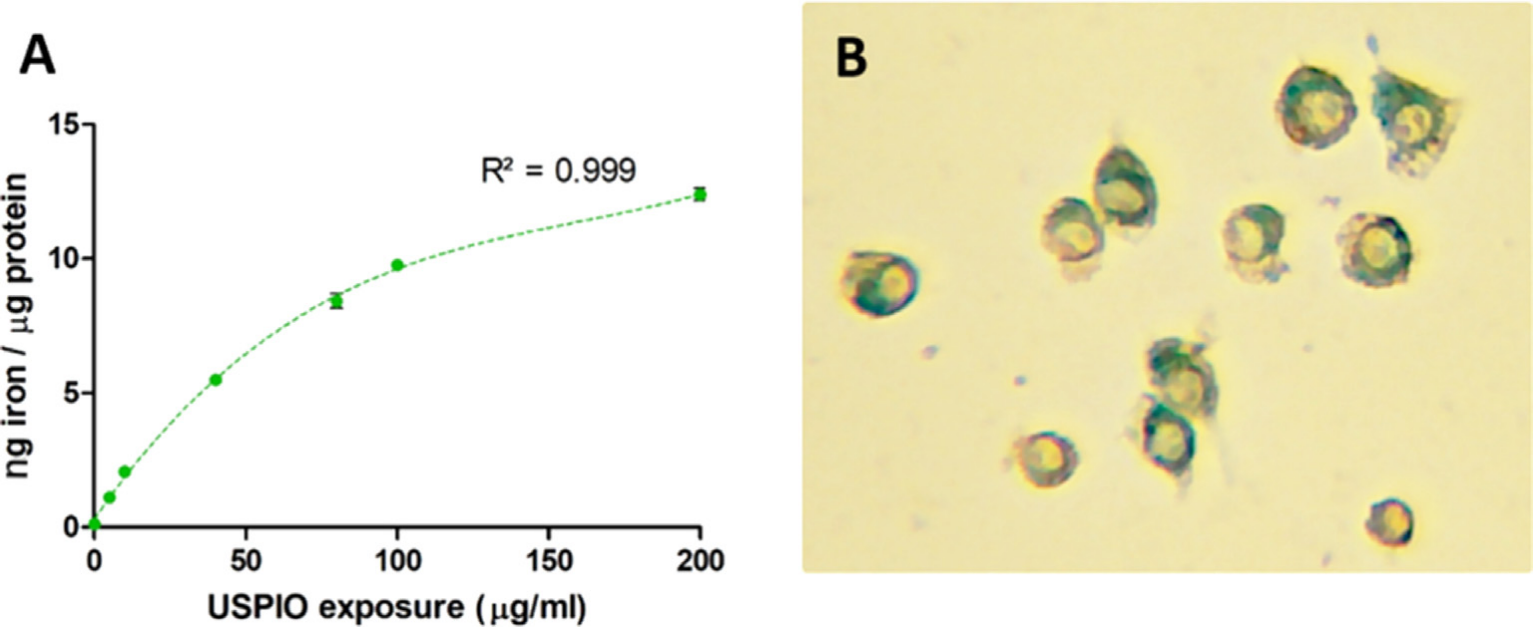
A: Iron quantification (from phagocytosed USPIO) using a 2,2′-Bipyridine colorimetric assay as a standard reference. Cell iron uptake appears non-linear, best described by the 3rd order polynomial y = 2E-06x3 − 0.0009x2 + 0.1616x + 0.3092; R^2^ = 0.999. All points represent triplicate mean ± SEM. B: Prussian blue visualised in J774 macrophages exposed to 40 µg/ml ferumoxytol USPIO and Perl stain; ×400 magnification light microscopy. (For interpretation of the references to colour in this figure legend, the reader is referred to the web version of this article.)

Table 1 shows the mean FFC-NMR derived average *R*_1_ across the entire magnetic field tested for non-fixed and fixed cells, the mean cytometry-derived non-fixed cell SSC and the mean cytometry-derived USPIO area/total cell area for both non-fixed and fixed cell suspensions, at all tested USPIO exposure concentrations. The detection range (0.115 ± 0.118 ng/µg to 12.398 ± 0.233 ng/µg protein) of the colorimetric iron quantification assay of non-fixed cells is shown for reference (*p* < 0.001 vs. control at all concentrations) as the ‘absolute’ intracellular iron amount (basal cellular iron content and phagocytosed USPIO iron content) against which all comparisons were made. The uptake of USPIO by J774 cells is modelled in a curvilinear fashion, as detected by this technique (Fig. 2). As seen in Table 1, all methods employed demonstrate ability to detect incremental levels of USPIO exposure which was quantified as intracellular iron.

**Table 1:** Summary iron quantification with FFC-NMR, imaging flow cytometry and a conventional colorimetric assay; SSC = side-scattered light; *p < 0.05 vs. control; **p < 0.001 vs. control. ‘Mean *R*1′ was obtained by averaging *R*1 values at all tested evolution fields for each USPIO exposure concentration (the average of all the R1 values on the dispersion curve for each tested exposure). The points on each dispersion curve were in themselves plotted using an average of 3 scans (each evolution field has 3 *R*1 values).

USPIO concentration (μg/ml medium)	FFC-NMR non-fixed Mean *R*_1_ of 1 × 10^6^ cell suspension (s^−1^)	FFC-NMR fixed Mean *R*_1_ of 1 × 10^6^ cell suspension (s^−1^)	Imaging cytometry non-fixed		Imaging cytometry fixed	Colorimetric reference iron assay Iron concentration of non-fixed 1 × 10^6^ cell culture (ng/μg protein)
			Mean USPIO area/cell area	Geometric mean SSC intensity	Mean USPIO area/cell area	
0 (control)	0.308 ± 0.014	0.439 ± 0.075	0.034 ± 0.001	26,860	0.044 ± 0.0004	0.115 ± 0.118
5	0.356 ± 0.013**	0.719 ± 0.026**	0.036 ± 0.001	32815**	0.046 ± 0.0003**	1.121 ± 0.045*
10	0.432 ± 0.016**	1.153 ± 0.024**	0.037 ± 0.001*	39573**	0.055 ± 0.0003**	2.074 ± 0.084*
40	0.706 ± 0.021**	2.348 ± 0.017**	0.069 ± 0.001**	69285**	0.072 ± 0.0003**	5.496 ± 0.134*
80	1.174 ± 0.031**	3.502 ± 0.147**	0.085 ± 0.001**	80967**	0.077 ± 0.0004**	8.421 ± 0.269*
100	1.239 ± 0.033**	5.174 ± 0.168**	0.090 ± 0.001**	82693**	0.078 ± 0.0003**	9.771 ± 0.100*
200	1.599 ± 0.041**	7.755 ± 0.257**	0.097 ± 0.001**	86373**	0.151 ± 0.0007**	12.398 ± 0.233*

### 3.1 Fast field-cycling nuclear magnetic resonance detection of USPIO

**Fig. 3:**
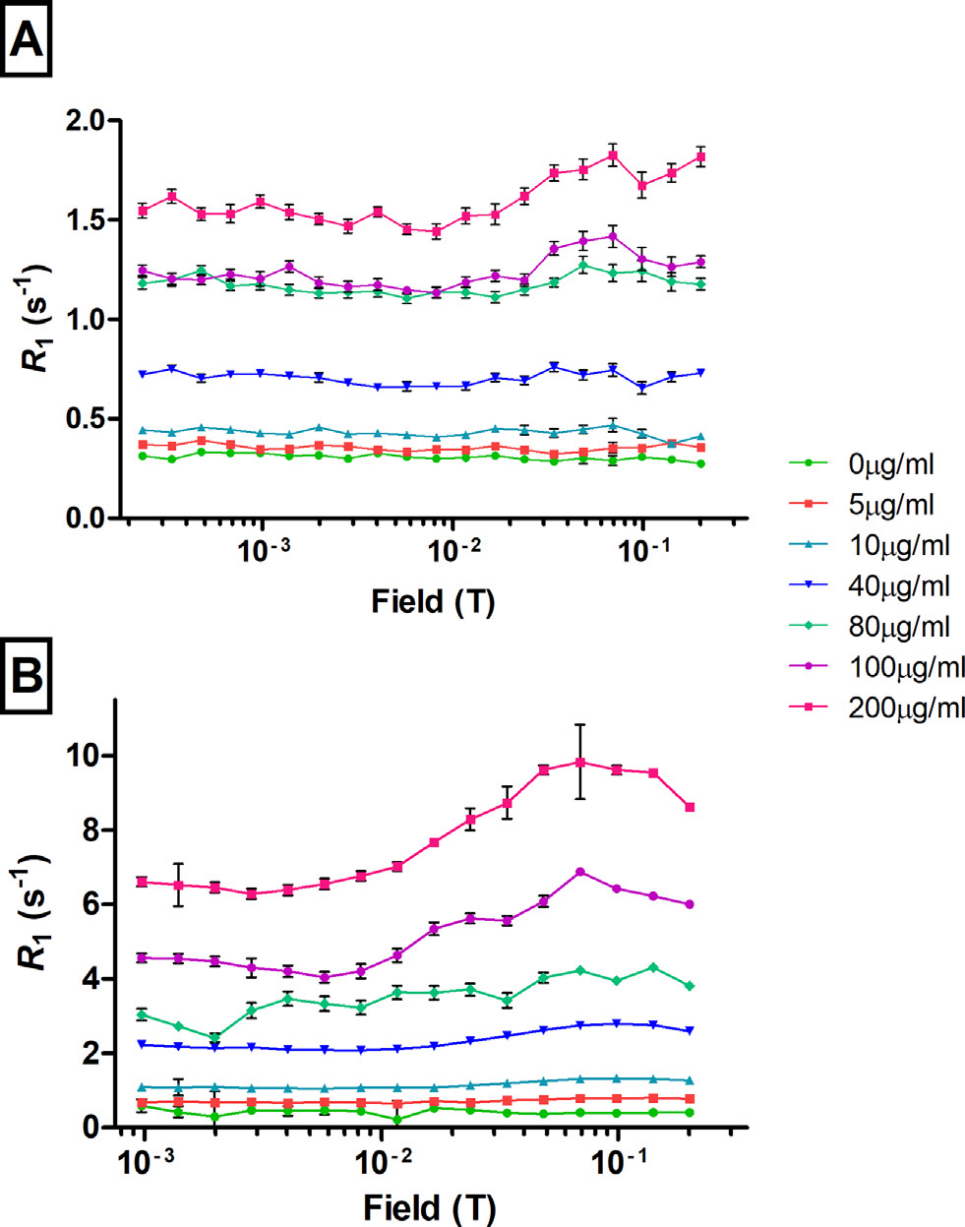
*R*_1_ dispersion curves of non-fixed (A) and fixed (B) J774 cell suspensions exposed to 0–200 µg/ml USPIO. Each data point represents a mean of triplicates ± SEM.

USPIO was detected with excellent resolution at all concentrations tested (*p* < 0.001 vs. control). There was good separation of the average *R*_1_ NMR dispersion profiles of both non-fixed (Fig. 3A) and fixed (Fig. 3B) USPIO-incubated cells compared to control, over the range of field strengths used in the FFC-NMR sequence and for all the USPIO exposure concentrations investigated. The *R*_1_ dispersion curves obtained from FFC-NMR in fixed cells showed the typical USPIO profile, with a peak at fields above 10 mT and a tail below (Fig. 3B); the position of the peaks appeared to shift slightly towards lower magnetic fields in non-fixed compared to fixed cells. There was strong positive correlation with colorimetric assay (*r* = 0.993 *p* < 0.001 non-fixed; *r* = 0.968 *p* = <0.001 fixed).

**Fig. 4:**
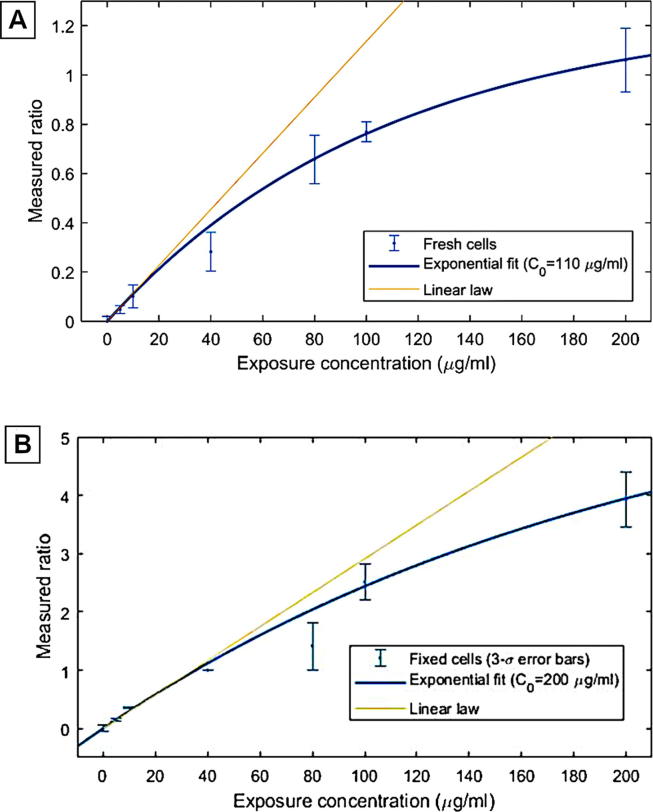
Effect of increasing macrophage USPIO exposure concentration on the average relaxivity of the USPIO compared to a reference NMRD profile in fresh (A) and fixed (B) cells. The average ratio between the NMRD profile and the reference profile shows that the relaxivity of internalised USPIO saturates with concentration, which is expected, but this effect is almost twice as important in fresh cells.

Both fresh and fixed cells exhibited USPIO relaxation that increased non-linearly with the particle concentration (Fig. 4A and B, respectively). The mono-exponential model used appeared to be a good approximation of the behaviour observed and provided a characteristic concentration of 108 (sigma = 25) and 210 (sigma = 140) µg/ml for fresh and fixed cells respectively. The amplitude of the relaxation contribution due to USPIO also increases significantly with cell fixation, turning from 1.26 (sigma = 0.14) to 6.4 (sigma = 3.2).

### 3.2 Imaging flow cytometry detection of USPIO

**Fig. 5:**
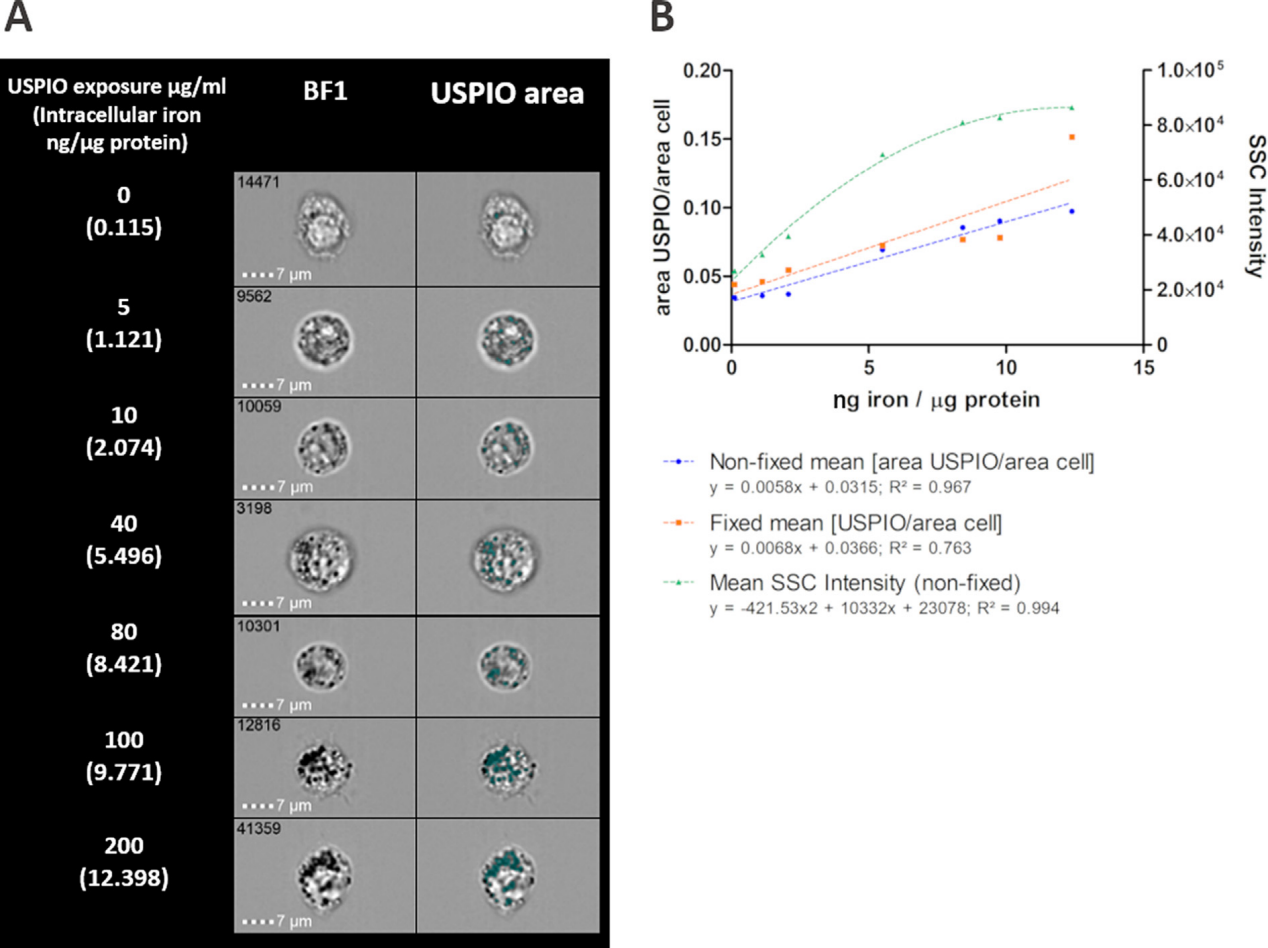
Imaging flow cytometric analysis of USPIO-exposed non-fixed and fixed cells. A: Macrophage imaging and localisation of intracellular USPIO aggregates (highlighted in green). B: The relative USPIO-occupied cell area as calculated by custom-generated image masks, and SSC intensity reflecting cell granularity (expressed as mean ± SEM), both increasing with higher USPIO exposure, based on intracellular iron amounts quantified colorimetrically (µg iron/ µg protein). Regression lines shown for all three. (For interpretation of the references to colour in this figure legend, the reader is referred to the web version of this article.)

Subjectively, more USPIO-containing cells were noted with increasing USPIO exposure in BF images, with increasing capture by the USPIO area mask (Fig. 5A). Mean USPIO area/cell area for both non-fixed and fixed cells incremented with increasing USPIO exposure, with sensitivity for detecting USPIO at exposures ≥10 µg/ml for non-fixed and ≥5 µg/ml for fixed cells (Table 1). There was strong positive correlation with colorimetric assay (r = 0.983, p < 0.001 non-fixed; r = 0.874, p < 0.05 fixed). Although the trends were similar, there was a small but significant difference in the detectable fraction of USPIO-occupied cell area with fixation, with an overall increase of this parameter at all concentrations inclusive of control samples (mean difference 20.2% ± 10.3% [range −13.4% to 55.6%]; p < 0.001 for all). Best fit was achieved using a simple linear regression model (*R*^2^ = 0.763 non-fixed; 0.967 fixed; Fig. 5B). In non-fixed cells SSC intensity reliably detected USPIO relative to control cells at exposures ≥5 µg/ml (p < 0.001 for all concentrations). This also correlated positively with colorimetric assay (*r* = 0.967 *p* < 0.001) but best fit could be expressed with a curvilinear model (*R*^2^ = 0.994; Fig. 5B).

## 4 Discussion

### 4.1 Key findings

The main finding of this proof-of-concept work shows that FFC-NMR can detect and quantify USPIO contrast using *T*_1_ dispersion information. FFC-NMR is capable of detecting cellular iron content at approximately 1.12 ng/µg cell protein (at the lowest tested exposure of 5 µg/ml USPIO) and resolves this from a control cell lysate that had not been exposed to USPIOs. These large *T*_1_ differences seen in the low magnetic field domain may allow us to exploit efficient *T*_1_-based contrast through the use of a gradient echo pulse sequence with FFC-MRI, since FFC enables “tuning” of the pulse sequence to specific features in the relaxivity curve, by suitable choice of the evolution field.

### 4.2 The effects of chemical fixation

The 5-fold increase observed in the USPIO contribution to the relaxation rate in fixed vs. fresh cells, may be attributed to the enhanced access of water molecules to sites of USPIO relaxation, since formaldehyde fixation is known to alter cell membranes, increasing their porosity and facilitating water exchange with the extracellular medium. Formaldehyde fixation has therefore increased the sensitivity of USPIO detection in our experiment. The non-linearity of the USPIO contribution with its concentration is likely due to aggregation of the nanoparticles in cells, which tends to quench its effect. This effect seems less pronounced in fixed cells, which improved the detection range of USPIO by FFC-NMR. Strijker et al. [31] described a three-compartment model for *T*_1_-relaxation time constant of systems with internalised paramagnetic contrast agents, employing a voxel containing an extracellular, cytoplasmic and vesicular compartment (with particular focus on the subcellular compartment). Their calculations mirrored those demonstrated experimentally. The multiexponential *T*_1_ relaxation and non-linear contrast agent dependence of *R*_1_ for contrast agent-loaded-cells was explained by relaxivity-‘quenching’, observed when contrast agents are internalised due to limited water exchange consequent to contrast agent entrapment in subcellular compartments.

Fixation on the other hand causes major changes in tissue biophysical properties. It reduces ADC_ex_ (the apparent diffusion coefficient of water in the extracellular space) and increases mean intracellular residence time (Ƭ = 1/*k*_ie_) [32]. Formaldehyde itself forms hydrates in aqueous solution that cross link a portion of the molecule into a polymeric matrix, slowing molecular motion and reducing water ADC and proton *T*_2_. Interaction with aquaporin channels has also been suggested, altering water mobility [32].

### 4.3 The effects of USPIO clustering

Clustering also affects USPIO relaxivity and is important to consider when interpreting results. In early clinical experiments utilising SPIO assessing hepatic and splenic uptake on MRI, *T*_1_ and *T*_2_ increased linearly with increasing SPIO exposures in agar gel phantoms, with similar effects quantified on clinical imaging (fast SE for *T*_2_, SPGR for *T*_1_, SPGR for *T*_2_*) [33]. The effect of clustering was more important for T2* relaxation effect caused by SPIO than for homogenous biodistribution. *T*_1_ was more pronounced in spleen where clusters are smaller, and *T*_2_* signal dropout smaller due to the same. Water molecules in the spleen can diffuse in the vicinity of SPIO clusters more frequently than in the liver, due to the larger number and smaller size of SPIO clusters.

Simon et al. [34] demonstrated that USPIO compartmentalisation by monocytes changes proton relaxivity, with higher *T*_1_ and *T*_2_ values of free extracellular USPIO in Ficoll solution at both 1.5 T and 3 T. At 3 T however, differences in *T*_1_ relaxivities of USPIO were smaller than 1.5 T, while differences in *T*_2_ were similar. Increasing *R*_1_ with USPIO dose was attributed to increasing interaction between iron oxide and water, while increased *R*_2_ due to decrease in water coefficient.

The non-linear relationship observed between relaxation rate and USPIO exposure however will have important implications for being able to separate signals from USPIO remaining in the extracellular space, from those internalised within phagocytic macrophages [34], as the motion of water protons differs significantly in the extracellular versus intracellular milieus [35], [36]. Previously published data reports that USPIO in solution has higher *T*_1_ and *T*_2_ than cells with completely internalised USPIO [34], [37].

### 4.4 Intracellular USPIO and flow cytometry

Whilst imaging flow cytometry detection by traditional side scatter was comparable in sensitivity to FFC-NMR, the USPIO-occupied area was only sensitive at exposures ≥10 µg/ml USPIO in non-fixed cells. Although imaging flow cytometry is not a conventional technique for iron quantification, it does uniquely provide quantifiable data on cell complexity [38], [39] (reflected in the increasing SSC with higher USPIO exposures) as well as imaging data demonstrating an increase in quantifiable dark locules. It also reassuringly demonstrated the internalisation of the USPIOs by macrophages *in vitro*. While this provided an excellent verification of the increase in cellular uptake of USPIOs in intact cells, the level of sensitivity is inferior to the FFC-NMR method and imaging cytometry cannot be translated into *in vivo* methods to detect inflammation.

### 4.5 Justification for the use of T_1_ and low magnetic fields

Early work assessing post-mortem samples of pathologically iron-overloaded (majority with beta thalassemia major) human myocardium *ex-vivo* with MR relaxometry, studied tissue containing much higher concentrations of iron [40]. Here myocardial iron concentration ranged between 0.18 and 53.4 mg/g dry weight and expressed a curvilinear relationship with *T*_2_*. Gradient echo *T*_2_* multiecho techniques can indeed rapidly assess for iron overload *in vivo* in transfusion-dependent beta thalassemia patients [41]. These techniques however are useful for identifying and monitoring frank iron overload syndromes [42]. Normal myocardial iron concentration have been reported to be 0.34 mg/g dry weight (range 0.29 – 0.47 mg/g). This raises an important consideration for the required sensitivity of NMR relaxometric or imaging methods in discerning differences between normal and USPIO-loaded inflamed tissue, as well as the localising ability of these techniques which is relevant to focal pathologies like coronary artery disease.

The limitations of analysing pixel maps of *T*_1_ and *T*_2_ are well recognized [43], [44], [45]. *T*_2_* relaxometry for quantitative analysis is generally limited by large scale field inhomogeneity with potential signal loss and *R*_2_* over-estimation. Very short *T*_2_* values may be error prone and shorter inter-echo times can also over-estimate *R*_2_*. *T*_2_* negative-signal imaging is associated with artefacts at the blood-pool to myocardium interface which need careful exclusion when selecting myocardial regions of interest. Blooming artefacts also commonly arise from nearby organs with high iron uptake, or from blood pool iron [46]. The reversible relaxation rate *T*_2_* is usually measured to detect compartmentalized SPIOs sensitively [47]. *T*_2_* relaxation rate is however also influenced by macroscopic susceptibilities that arise from air-tissue interfaces (e.g. lung-liver interface). These susceptibilities enhance signal decay, increasing linearly with the field strength leading to overestimated relaxation rates or the obscuring of small concentrations of SPIO-labelled [48]. The very short *T*_1_ of USPIO-associated tissue has been a significant deterrent in its clinical use for USPIO quantification at conventional fixed fields. A number of positive contrast techniques relying on pulse sequence modifications have been described however [49].

*T*_2_ has limitations in detecting low iron concentrations where *T*_2_ or *T*_2_* durations are too short to be imaged. Newer techniques such as sweep imaging with Fourier transformation (SWIFT) [50] may overcome this limitation as demonstrated at 9.4 T in the detection of SPIO-labelled embryonic stem cells injected into murine hearts. Broadband frequency‐swept excitation and extremely short acquisition delay are unique features of SWIFT which make it particularly well suited for imaging frequency‐shifted resonances and for minimizing signal loss from *T*_2_*‐induced dephasing.

Whereas high field aims to improve image resolution and increase SNR, on the other hand cost, magnet size, tissue heating and safety are major concerns. Versluis et al. [51] report generally good tolerance at 7 T human scans with 3% of volunteers reporting discomfort, most commonly dizziness while moving into/out of the magnet, high noise levels and perception of a metallic taste. In addition, higher magnetic fields invite more artefact particularly from metallic prostheses. There is increased chemical shift artefact, higher specific absorption rate (SAR), *B*_1_ inhomogeneity and spatial variation of flip angles [52].

Stroh et al. [53] highlighted some of these limitations when monitoring the biodistribution of very small iron oxide nanoparticle-laden (VSOP, 9 nm diameter, smaller than USPIO) grafted stem cells *in vivo* (stereotactically injected into rat striatum). *T*_2_* signal was detected from approximately 1000 grafted VSOP-loaded cells in transplantation sites but this was performed at 17.6 T – not currently possible in humans. The proposed advantage of the high field is the higher equilibrium magnetisation giving higher intrinsic SNR, with changes in magnetic susceptibility of iron particles leading to more pronounced signal reduction in the grafted cells than at lower field strengths. The caveat was the high sensitivity to artefact particularly when remedied with steady-state free precession (SSFP) techniques in inhomogenous media or if the object was moving. A low magnetic field was proposed to be adequate however for the detection of the bulk motion of several thousand cells.

Low fixed magnetic field clinical imaging is gaining considerable interest at present [54] due to potential cost-effectiveness. The reduced SAR and susceptibility effects resulting from the use of a lower magnetic field has clear application for imaging in the vicinity of metallic implants. This can translate into higher patient safety and clinical utility – notably reducing the hazard from the ‘missile effect’ and RF heating. The reduced heating/SAR would allow higher flip angles to maximise CNR. Low field MRI is also much less sensitive to chemical shift artefacts [26]. The above strengths are currently paving the way for the development of cardiac MR-guided interventional devices [55].

### 4.6 Justification for the use of FFC-MR

FFC-MR advantageously increases the available frequency (field) range permitting faster scanning, as the detection field is constant, and without the RF apparatus requiring adjustment for a varying relaxation field. Systematic errors in *T*_1_ caused by non-homogenous *T*_1_ RF fields are reduced as the perturbation of the sample occurs by a rapid jump of the external field *B*_0_ and not by an RF pulse.

*T*_1_ measurements on conventional, fixed-field NMR or MRI devices can only report on a single characteristic time of molecular motion. FFC however allows access to several decades of motional dynamics [56], typically from tens of nanoseconds to several milliseconds. Measurements of *T*_1_ dispersion with the magnetic field strength, allow quantitative insight into the nature of molecular motion and are exploited to determine structural [57], [58], [59]. These time scales correspond to structures ranging in size from tens of nanometres up to several micrometres, highly relevant to the study of biological tissues. For example, the quadripolar dip observed in FFC-MR *T*_1_-dispersion curves is a consequence of cross-relaxation from dipole to quadripole nuclei, and is inaccessible by conventional MRI [60], [61], [62]. This can translates to the study of large molecules and protein structures e.g. in thrombus and cartilage [25], [63].

We have recently reported on the first human size FFC-MR prototype in the world which will be well suited for such clinical applications. This breakthrough in MRI technology can access magnetic fields from 50 μT to 0.2 T, thereby exploring almost four decades of molecular-dynamics time scales, from 0.5 ms to 100 ns.

Experiments on phantoms using our FFC-MR prototype successfully allowed differentiation between cross-linked bovine serum albumin (BSA) and manganese chloride solutions simply by clustering over *T*_1_ dispersion information [24] shapes of the dispersion curves provided by the two compounds. Determining sample concentration was also possible using the average T1 value. The relationship between the linearity of the dispersion amplitude and the concentration of relaxing agents demonstrated that FFC-MRI can differentiate materials easily as well as provide quantitative information about their composition. Accurate characterisation of the dispersion of biological tissues could lead to quantitative mapping of tissue-specific *T*1. Furthermore dispersion curves may function as ‘biomarkers’ for certain pathologies and resolve differences in tissue not detected by conventional MRI. Previous work by our group and others [25], [26], [60], [61], [63], [64], [65], [66], [67], [68], [69], [70], [71] investigating tissue biopsies, showed potential biomarkers in a variety of pathologies that can now be investigated *in vivo*, using endogenous contrast FFC-MRI technology therefore has major potential to complement conventional MRI scanners in clinical diagnostics. Clinical imaging using FFC-MR has already been demonstrated in a study of acute stroke [72]. The current work explores a further FFC-MR avenue: the role of exogenous USPIO-based contrast, as proof-of-principle and stepping stone for the MR/MRI study of inflammation *in vivo*.

### 4.7 Study limitations

The FFC-NMR scanners could only analyse one sample at a time and therefore the staggering of fresh cell suspensions could have affected cell viability and possibly influenced *T*_1_ relaxation time. To mitigate this, the samples were refrigerated at 4 °C before their scan to minimise any such possibility pending scanning. Cell viability was only confirmed with light microscopy. It is possible that a degree of cell lysis with the passage of time could have resulted in extracellular, free USPIO, which may have affected FFC-NMR *T*_1_ dispersion data. The imaging capability of our cytometric technique, however, provided an additional check of the integrity of cell membranes in both non-fixed and fixed samples.

The FFC-MRI scanner can exhibit relatively large field instabilities during operation, com-pared to fixed-field MRI. Nevertheless, post-acquisition corrections can be used to obtain clinically robust images from *T*1 dispersion data [73]. Our future work endeavours to develop FFC-MR *in vivo* assessment and imaging utilising USPIO.

## 5 Conclusion

In this work we used a cell-based model for the *in vitro* detection of intra-cellular USPIO by FFC-NMR as a proof of principle for future translation towards imaging using FFC-MRI. We demonstrated for the first time that FFC-NMR is capable of quantitative assessment of intra-cellular USPIO, with levels of detection directly relevant to biological applications. The use of FFC-MR may complement clinical diagnostics by accessing relaxometry features not readily available at higher field strengths or in fixed fields. Further work is required to develop this technology for the *in vivo* assessment of inflammation and/or unlock its imaging potential with USPIO.

## Funding

DKD received funding from British Heart Foundation Project Grant PG/15/108/31928. DJL received funding from the European Commission – ‘Improving Diagnosis by Fast Field-Cycling MRI’ grant number 668119.

## Ethical approval

This article does not contain any studies with human participants or animals performed by any of the authors.

## Author contributions

HA – Conceptualisation. Lead role in overall experiment design. Lead role conducting FFC-MR and colorimetric experiments. Contributory role conducting Flow Cytometry experiments. Lead role in manuscript authorship.

LB – Lead role in developing FFC-MR technology. Equal role in conducting FFC-MR experiments. Contributor to manuscript authorship and editing.

AE – Contributing role to Flow Cytometry experiment design. Lead role in conducting Flow Cytometry experiments. Contributor to manuscript authorship.

DL – Contributing role to Flow Cytometry experiment design. Lead role in conducting Imaging Flow Cytometry experiments.

RY - Lead role to Flow Cytometry experiment design. Contributing role in conducting Imaging Flow Cytometry experiments. Contributor to manuscript authorship. Supervisory role in all Flow Cytometry work.

PJR – Lead role in developing FFC-MR technology. Contributing role to conducting FFC-MR experiments.

LC – Contributing role to calibrating colorimetric experiments.

HMW - Contributing role to colorimetric and Flow Cytometry experiment design. Supervisory role in all tissue culture work. Contributor to manuscript authorship.

DJL - Lead role in developing FFC-MR technology. Contributor to manuscript authorship.

DKD – Generated the concept of increased accuracy of detection of iron at low field. Contributory role in overall experiment design. Contributory role in manuscript authorship. Supervisory role for project.

## Declaration of Competing Interest

DJL, DKD, HMW, RY, LC, PJR, LB, AE, DL and HA have no conflicts of interest to declare.
